# Supplementary material for: Genetic liability to schizophrenia is associated with exposure to traumatic events in childhood
Source: Psychol Med. 2020 Apr 1;51(11):1814–21. doi: 10.1017/S0033291720000537 (PMC8381289; doi:10.1017/S0033291720000537)
Supplement: Supplementary file 1 [file S0033291720000537sup001.docx]

**Supplementary information**

Table of contents

[ALSPAC genetic data generation and quality control 2](#_Toc31646349)

[The Norwegian Mother, Father and Child Cohort Study (MoBa) 4](#_Toc31646350)

[MoBa genetic data generation and quality control 5](#_Toc31646351)

[ALSPAC imputation strategy 7](#_Toc31646352)

[Table S1. Unadjusted association between all schizophrenia PGS at all thresholds and trauma measured at age 0-4.9 years in ALSPAC 8](#_Toc31646353)

[Table S2. Prevalence of trauma across each age range and trauma domain among ALSPAC participants 9](#_Toc31646354)

[Table S3. Proportion of children in the Norwegian Mother, Father and Child Cohort Study (MoBa) with maternally reported traumatic experience at 8 years 10](#_Toc31646355)

[Table S4. Unadjusted association between schizophrenia PGS_0.05_ and any trauma in ALSPAC – complete cases 11](#_Toc31646356)

[Table S5. Unadjusted association between schizophrenia PGS_0.05_ and subtypes of trauma across childhood and adolescence (age 0-17 years) in ALSPAC – complete cases 11](#_Toc31646357)

[Table S6. Unadjusted association between alternative PGS_0.05_ and childhood trauma between age 0-4.9 years in ALSPAC 12](#_Toc31646358)

# ALSPAC genetic data generation and quality control

ALSPAC children were genotyped using the Illumina HumanHap550 quad chip genotyping platforms. The resulting raw genome-wide data were subjected to standard quality control methods. Individuals were excluded on the basis of gender mismatches; minimal or excessive heterozygosity; disproportionate levels of individual missingness (>3%) and insufficient sample replication (IBD < 0.8). Population stratification was assessed by multidimensional scaling analysis and compared with Hapmap II (release 22) European descent (CEU), Han Chinese, Japanese and Yoruba reference populations; all individuals with non-European ancestry were removed. SNPs with a minor allele frequency of < 1%, a call rate of < 95% or evidence for violations of Hardy-Weinberg equilibrium (P < 5E-7) were removed. Cryptic relatedness was measured as proportion of identity by descent (IBD > 0.1). Related subjects that passed all other quality control thresholds were retained during subsequent phasing and imputation. 9,115 subjects and 500,527 SNPs passed these quality control filters.

ALSPAC mothers were genotyped using the Illumina human660W-quad array at Centre National de Génotypage (CNG) and genotypes were called with Illumina GenomeStudio. PLINK (v1.07) was used to carry out quality control measures on an initial set of 10,015 subjects and 557,124 directly genotyped SNPs. SNPs were removed if they displayed more than 5% missingness or a Hardy-Weinberg equilibrium P value of less than 1.0e-06. Additionally, SNPs with a minor allele frequency of less than 1% were removed. Samples were excluded if they displayed more than 5% missingness, had indeterminate X chromosome heterozygosity or extreme autosomal heterozygosity. Samples showing evidence of population stratification were identified by multidimensional scaling of genome-wide identity by state pairwise distances using the four HapMap populations as a reference, and then excluded. Cryptic relatedness was assessed using a IBD estimate of more than 0.125 which is expected to correspond to roughly 12.5% alleles shared IBD or a relatedness at the first cousin level. Related subjects that passed all other quality control thresholds were retained during subsequent phasing and imputation. 9,048 subjects and 526,688 SNPs passed these quality control filters.

After combining genotype data in the mothers and the children, SNPs with genotype missingness above 1% were removed due to poor quality (11,396 SNPs removed) and a further 321 subjects were removed due to potential ID mismatches. This resulted in a dataset of 17,842 subjects. Imputation of the target data was performed using Impute V2.2.2 against the 1000 genomes reference panel (Phase 1, Version 3) (all polymorphic SNPs excluding singletons), using all 2186 reference haplotypes (including non-Europeans).

This gave 8,237 eligible children and 8,196 eligible mothers with available genotype data after exclusion of related subjects using cryptic relatedness measures described previously.

3,453 ALSPAC mother and fathers and 535,478 SNPs were genotyped using the Illumina HumanCoreExome chip genotyping platforms by the ALSPAC lab and called using GenomeStudio. The resulting raw genome-wide data were subjected to standard quality control methods using PLINK (v1.07). Individuals were excluded on the basis of gender mismatches (n = 80); minimal or excessive heterozygosity (n = 64); disproportionate levels of individual missingness (>5%, n = 60) and possible contamination (n = 3). Population stratification was assessed by multidimensional scaling analysis and compared with 1000 Genomes phase 3 data and principal component analysis (n = 266); all individuals with non-European ancestry were removed. Cryptic relatedness was measured as SNP relatedness in GCTA (relatedness > 0.1, n = 69 removed). SNPs with a call rate of < 95% or evidence for violations of Hardy-Weinberg equilibrium (P < 1E-7) and those which failed GenomeStudio quality control measures were removed (n = 21,298). 6,594 duplicate SNPs were also removed.

Data was phased for 3074 samples that passed QC but contained related subjects in SHAPEIT v2.r837. The following were then removed: 155,336 monomorphic SNPs, 1033 markers not in 1000 genomes, 11,842 A/T or G/C SNPs and 10 duplicate sites to give 337,732 SNPs on chromosomes 1-23. Of the 329,363 markers on chromosomes 1-22, 298,742 overlapped the reference genome. These were imputed to the 1000 genomes phase 1 version 3 using the Michigan Imputation Server. 1722 eligible fathers remained after QC, exclusion of duplicate subjects and individuals who had withdrawal of consent.

# The Norwegian Mother, Father and Child Cohort Study (MoBa)

MoBa is a prospective population-based pregnancy cohort study conducted by the Norwegian Institute of Public Health (Magnus et al., 2016). Participants were recruited from all over Norway from 1999-2008. In 40.6 % of the pregnancies, women consented to participate. The cohort now includes 114,500 children, 95,200 mothers and 75,200 fathers. Blood samples were obtained from both parents during pregnancy and from mothers and children (umbilical cord) at birth. Genotyping is ongoing. The current study is based on version 12 of the quality-assured data files released for research in January 2019. The establishment and initial data collection in MoBa were previously based on a license from the Norwegian Data protection agency and approval from The Regional Committees for Medical and Health Research Ethics (REC); MoBa is now regulated by regulations related to the Norwegian Health Registry Act. The current analyses were approved by REC (reference number 2016/1702).

**MoBa genetic data generation and quality control**

Approximately 17,000 trios from the Norwegian Mother, Father and Child cohort were genotyped in three batches. Genotypes were called using GenomeStudio (Illumina, San Diego, USA) and converted to PLINK format files. The first batch, comprising 20,664 individuals and 542,585 SNPs was genotyped at the Genomics Core Facility (Iceland) using the Illumina HumanCoreExome (Illumina, San Diego, USA) genotyping array, version 12 1.1. The second batch, comprising 12,874 individuals and 547,644 SNPs was genotyped at the Genomics Core Facility (Iceland) using the Illumina HumanCoreExome (Illumina, San Diego, USA) genotyping array, version24 1.0. The third batch, comprising 17,949 individuals and 692,367 SNPs, was genotyped at ERASMUS MC (the Netherlands) using the Illumina Global Screening Array (Illumina, San Diego, USA) version 24 1.

PLINK version 1.90 beta 3.36 (<http://pngu.mgh.harvard.edu/purcell/plink/>) was used to conduct the quality control, which has previously been described by Helgeland et al (2019). Known problematic SNPs previously reported by the Cohorts for Heart and Aging Research in Genomic Epidemiology (CHARGE) consortium and Psychiatric Genomics Consortium (PGC) were excluded from each batch. Duplicate samples were removed, and each genotyping batch was split into parents and offspring. Quality control was then conducted by genotyping array in parents and offspring separately.

Individuals were excluded if they had a genotyping call rate below 95% or autosomal heterozygosity greater than four standard deviations from the sample mean. SNPs were excluded if they were ambiguous (A / T and C / G), had a genotyping call rate below 98%, minor allele frequency of less than 1%, or Hardy-Weinberg equilibrium P-value less than 1 × 10^-6^. Population stratification was assessed, using the HapMap phase 3 release 3 as a reference, by principal component analysis using EIGENSTRAT version 6.1.4. Visual inspection identified a homogenous population of European ethnicity and individuals of non-European ethnicity were removed. Individuals with a genotyping call rate below 98% or autosomal heterozygosity greater than four standard deviations from the sample mean were then removed. A sex check was done by assessing the sex declared in the pedigree with the genetic sex, which was imputed based on the heterozygosity of chromosome X. When sex discrepancies were identified, the individual was flagged. Relatedness was assessed by flagging one individual from each pairwise comparison of identity-by-descent with a pi-hat greater than 0.1.

The parents and offspring datasets were then merged into one dataset per genotyping batch; keeping only the SNPs that passed quality control in both datasets. All individuals passing the genotyping call rate and autosomal heterozygosity measures were included in the merged datasets. Therefore, the merged datasets included individuals previously excluded or flagged as a duplicate, ethnic outlier, having a sex discrepancy, or high level of relatedness. Concordance checks were then conducted on validated duplicates. Duplicate, tri-allelic and discordant (any discordance between the validated duplicates) SNPs were excluded. Individuals and SNPs with a genotyping call rate below 98% in the merged datasets were excluded. The duplicate sample that was removed before the start of the quality control was then excluded. Mendelian errors identified by the assessment of duos and trios were then recoded to missing. Insertions and deletions were also excluded.

After QC the Human Core Exome 12 batch comprised 20,231 individuals and 384,855 SNPs, the Human Core Exome 24 batch comprised 12,757 individuals and 396,189 SNPs, and the Global Screening Array batch comprised 17,742 individuals and 568,275 SNPs. Phasing was conducted using Shapeit 2 release 837 and the duoHMM approach was used to account for the pedigree structure. Imputation was conducted using the Haplotype reference consortium (HRC) release 1-1 as the genetic reference panel. The Sanger Imputation Server was used to perform the imputation with the Positional Burrows-Wheeler Transform (PBWT). The phasing and imputation were conducted separately for each genotyping batch.

Post imputation quality control was performed by initially converting the dosages to best-guess genotypes. Individuals were removed if they had a genotyping call rate less than 99% or were of non-European ethnicity. SNPs with an imputation INFO quality score less than 0.8, genotyping call rate less than 98%, minor allele frequency less than 1%, or a Hardy-Weinberg equilibrium P-value less than 1 × 10^-6^ were removed. After quality control, a core homogeneous sample of European ethnicity (based on PCA of markers overlapping with available HapMap markers), unrelated (within generation, defined as accumulated identity-by-descent <0.015 and overall identity-by-descent PI_HAT <10%) individuals across all batches and arrays were available for use in analysis (N_children_ = 15,208; N_mothers_ = 14,804; N_fathers_ = 15,198).

***References***

Helgeland, Ø., Vaudel, M., Juliusson, P.B. et al. Genome-wide association study reveals dynamic role of genetic variation in infant and early childhood growth. Nat Commun 10, 4448 (2019). <https://doi.org/10.1038/s41467-019-12308-0>.

# ALSPAC imputation strategy

For the ALSPAC cohort, the complete sample with data on polygenic scores and trauma data ranged between 4,718 and 6,306. To address potential bias from attrition, we conducted multiple imputation using the ICE command implemented in Stata 15 (StataCorp, 2017) and followed the imputation strategy described by Croft and colleages (2018). 50 imputed datasets were created using information from variables included in our analyses and additional information from 49 variables associated with observed data and missing-ness that would make the missing at random assumption more plausible. The following variables were included in the multiple imputation model: highest maternal education, marital status, perinatal depression, drug use, domestic violence, partner affection and aggression, maternal age at delivery, ethnicity, child temperament, residency (rented or mortgaged), mother’s reported alcohol consumption at 6 months old, mother’s reported smoking during pregnancy, social class, mother’s life events score, borderline personality disorder assessment at 11 years based on the UK Childhood Interview for DSM-IV BPD, any ICD-10 or DSM-IV diagnosis at 10 years using the Development Well-Being Assessment (DAWBA), symptoms of depression and anxiety measured at 13 years old using the Moods and Feelings Questionnaire (MFQ); self-reported alcohol consumption at 13 years and smoking frequency at 13 years and self-report measures of PLIKS completed at 12, 13, 14 and 16 years old. Predictive mean matching was used for non-normally distributed variables. The parameter estimates for each imputation were combined using Rubin’s rules as applied by the ‘mi estimate’ package in Stata.

***References***

Croft, J. et al. (2019) ‘Association of Trauma Type, Age of Exposure, and Frequency in Childhood and Adolescence With Psychotic Experiences in Early Adulthood’, JAMA Psychiatry. American Medical Association, 76(1), p. 79. doi: 10.1001/jamapsychiatry.2018.3155.

StataCorp. 2017. *Stata Statistical Software: Release 15*. College Station, TX: StataCorp LLC.

# Table S1. Unadjusted association between all schizophrenia PGS at all thresholds and trauma measured at age 0-4.9 years in ALSPAC

|  | Child PGS  (N=7,426) | | | | Mother PGS  (N=7,380) | | | Father PGS  (N=1,215) | | |  |
| --- | --- | --- | --- | --- | --- | --- | --- | --- | --- | --- | --- |
| PGS threshold | OR | 95% CI | P-value | OR | | 95% CI | P-value | OR | 95% CI | P-value | |
| 0.5 | 1.14 | 1.07,1.21 | 7.6x10^-6^ | 1.16 | | 1.09,1.22 | 7.6x10^-7^ | 1.13 | 0.98,1.31 | 0.094 | |
| 0.4 | 1.14 | 1.08,1.21 | 6.9x10^-6^ | 1.16 | | 1.09,1.23 | 4.4x10^-7^ | 1.14 | 0.98,1.31 | 0.087 | |
| 0.3 | 1.15 | 1.08,1.22 | 4.3x10^-6^ | 1.16 | | 1.09,1.23 | 3.4x10^-7^ | 1.14 | 0.99,1.32 | 0.070 | |
| 0.2 | 1.14 | 1.08,1.21 | 5.9x10^-6^ | 1.16 | | 1.09,1.23 | 3.2x10^-7^ | 1.15 | 1.00,1.34 | 0.052 | |
| 0.1 | 1.14 | 1.07,1.21 | 1.6x10^-5^ | 1.16 | | 1.11,1.23 | 1.7x10^-7^ | 1.13 | 0.98,1.31 | 0.097 | |
| 0.05 | 1.15 | 1.08,1.21 | 6.4x10^-6^ | 1.19 | | 1.12,1.25 | 1.8x10^-8^ | 1.13 | 0.97,1.30 | 0.118 | |
| 0.01 | 1.14 | 1.07,1.21 | 1.2x10^-5^ | 1.17 | | 1.11,1.23 | 9.6x10^-8^ | 1.11 | 0.95,1.28 | 0.181 | |
| 0.001 | 1.09 | 1.03,1.16 | 0.003 | 1.12 | | 1.05,1.17 | 1.7x10^-4^ | 1.07 | 0.92,1.25 | 0.349 | |
| 0.0001 | 1.07 | 1.02,1.14 | 0.010 | 1.09 | | 1.03,1.16 | 0.002 | 1.06 | 0.91,1.23 | 0.426 | |
| 1x10^-5^ | 1.09 | 1.03,1.16 | 0.002 | 1.09 | | 1.03,1.16 | 0.002 | 1.04 | 0.90,1.21 | 0.610 | |
| 1x10^-6^ | 1.07 | 1.01,1.14 | 0.015 | 1.07 | | 1.01,1.13 | 0.020 | 0.98 | 0.85,1.14 | 0.825 | |
| 1x10^-7^ | 1.08 | 1.02,1.14 | 0.008 | 1.06 | | 1.00,1.13 | 0.042 | 1.02 | 0.88,1.19 | 0.780 | |
| 5x10^-8^ | 1.09 | 1.03,1.15 | 0.002 | 1.05 | | 0.99,1.12 | 0.095 | 1.01 | 0.87,1.17 | 0.902 | |

OR: odds ratio; PGS: polygenic score; CI: confidence interval

# Table S2. Prevalence of trauma across each age range and trauma domain among ALSPAC participants

|  |  | Complete cases | | Imputed dataset | |
| --- | --- | --- | --- | --- | --- |
|  |  | N | % | N* | % |
| Any childhood trauma | 0-4.9 years | 8,079 | 24.5 | 9,946 | 27.2 |
|  | 5-10.9 years | 7,076 | 40.7 | 9,946 | 47.9 |
|  | 11-17 years | 6,087 | 36.2 | 9,946 | 46.3 |
|  | 0-17 years | 7,905 | 60.0 | 9,946 | 70.8 |
| Specific trauma domains | Bullying | 5,780 | 38.0 | 9,946 | 35.3 |
|  | Domestic violence | 5,431 | 34.4 | 9,946 | 27.6 |
|  | Sexual abuse | 3,353 | 13.5 | 9,946 | 10.3 |
|  | Emotional neglect | 5,011 | 10.7 | 9,946 | 9.8 |
|  | Emotional cruelty | 5,882 | 28.2 | 9,946 | 26.2 |
|  | Physical cruelty | 4,708 | 27.8 | 9,946 | 24.4 |

* This refers to individuals with *either* child or maternal genetic data available

# Table S3. Proportion of children in the Norwegian Mother, Father and Child Cohort Study (MoBa) with maternally reported traumatic experience at 8 years

| **Type of trauma** | **Question** | **Definition** | **Proportion*** |
| --- | --- | --- | --- |
| Bullying | Has your child been subjected to beating, kicking or other violence by other children in the past 12 months? | 1=Once in a while or more (2-3 times a month/Once a week/Several times a week) vs 0=Never | 10,049  (23.8%) |
| Emotional neglect | You let your child know when he/she is doing a good job at something? | 1=Never/Almost never/Sometimes vs 0=Often/Always | 805  (1.9%) |
| Physical abuse | Has your child been subjected to beating, kicking or other violence by adults in the past 12 months? | 1=Once in a while or more (2-3 times a month/Once a week/Several times a week) vs 0=Never | 297  (0.7%) |
| Any of the above |  |  | 10,682 (25.3%) |

***** Data on the three trauma questions was available on 42,236 children in MoBa

# Table S4. Unadjusted association between schizophrenia PGS_0.05_ and any trauma in ALSPAC – complete cases

|  | Child PGS_0.05_ | | | | Mother PGS_0.05_ | | | |
| --- | --- | --- | --- | --- | --- | --- | --- | --- |
| Age range | N | OR | 95% CI | P-value | N | OR | 95% CI | P-value |
| 0-4.9 years | 6,286 | 1.13 | 1.07,1.20 | 2.7x10^-5^ | 6,056 | 1.15 | 1.09,1.22 | 3.1x10^-6^ |
| 5-10.9 years | 5,736 | 1.04 | 0.99,1.10 | 0.104 | 5,329 | 1.06 | 1.00,1.12 | 0.038 |
| 11-17 years | 5,062 | 1.10 | 1.04,1.16 | 0.001 | 4,718 | 1.12 | 1.06,1.19 | 1.2x10^-4^ |
| 0-17 years | 6,306 | 1.09 | 1.03,1.14 | 0.001 | 5,918 | 1.07 | 1.01,1.12 | 0.015 |

OR: odds ratio; PGS: polygenic score; CI: confidence interval

# Table S5. Unadjusted association between schizophrenia PGS_0.05_ and subtypes of trauma across childhood and adolescence (age 0-17 years) in ALSPAC – complete cases

|  | Child PGS | | |  | Mother PGS | | |  | |  |
| --- | --- | --- | --- | --- | --- | --- | --- | --- | --- | --- |
| Type of trauma | OR | 95% CI | P-value | N | OR | 95% CI | P-value | | N | |
| Bullying | 1.05 | 0.99,1.11 | 0.135 | 4,826 | 1.01 | 0.95, 1.07 | 0.703 | | 4,446 | |
| Domestic violence | 1.10 | 1.03,1.17 | 0.003 | 4,390 | 1.19 | 1.12,1.27 | 1.3x10^-7^ | | 4,138 | |
| Sexual abuse | 1.20 | 1.08,1.33 | 0.001 | 2,822 | 1.17 | 1.04,1.31 | 0.007 | | 2,611 | |
| Emotional neglect | 1.09 | 0.99,1.20 | 0.093 | 4,269 | 1.12 | 1.01,1.24 | 0.033 | | 3,984 | |
| Emotional cruelty | 1.17 | 1.10,1.25 | 1.6x10^-6^ | 4,795 | 1.17 | 1.10,1.25 | 2.6x10^-6^ | | 4,488 | |
| Physical cruelty | 1.14 | 1.06,1.22 | 2.4x10^-4^ | 3,961 | 1.17 | 1.09,1.26 | 3.4x10^-5^ | | 3,682 | |

OR: odds ratio; PGS: polygenic score; CI: confidence interval

# Table S6. Unadjusted association between alternative PGS_0.05_ and childhood trauma between age 0-4.9 years in ALSPAC

|  | Discovery GWAS sample size | Child PGS  (N=7,426) | | | Mother PGS  (N=7,380) | | |
| --- | --- | --- | --- | --- | --- | --- | --- |
| PGS |  | OR | 95% CI | P-value | OR | 95% CI | P-value |
| ADHD | 20,183 cases, 31,191 controls | 1.09 | 1.03, 1.15 | 0.002 | 1.10 | 1.04, 1.17 | 0.001 |
| ASD | 18,381 cases, 27,969 controls | 1.03 | 0.97, 1.09 | 0.369 | 1.04 | 0.98, 1.10 | 0.175 |
| BIP | 29,494 cases, 169,118 controls | 1.07 | 1.01, 1.14 | 0.018 | 1.08 | 1.02, 1.14 | 0.007 |
| Cross Disorder | 33,332 cases, 27,888 controls | 1.13 | 1.07, 1.20 | 2.45x10^-5^ | 1.09 | 1.03, 1.16 | 0.002 |
| MDD | 135,458 cases, 344,901 controls | 1.16 | 1.10, 1.23 | 2.85x10^-7^ | 1.10 | 1.03, 1.16 | 0.002 |
| Neuroticism | 329,821 | 1.15 | 1.08, 1.22 | 4.33x10^-6^ | 1.17 | 1.10, 1.24 | 7.22x10^-8^ |
| Schizophrenia | 40,675 cases, 64,643 controls | 1.14 | 1.07, 1.20 | 1.36x10^-5^ | 1.13 | 1.06, 1.20 | 5.91x10^-5^ |

OR: odds ratio; PGS: polygenic score; CI: confidence interval
